# Supplementary material for: The Metabolomics Response of Solanum melongena L. Leaves to Various Forms of Pb
Source: Nanomaterials (Basel). 2023 Nov 8;13(22):2911. doi: 10.3390/nano13222911 (PMC10675538; doi:10.3390/nano13222911)
Supplement: Supplementary file 1 [file nanomaterials-13-02911-s001.zip › nanomaterials-2628727-supplementary.pdf]

Supplementary Materials

The Metabolomics Response of *Solanum melongena* L. Leaves to Various Forms of Pb

Siyu Zhang [1,†], Bing Zhao [1,2,†], Xuejiao Zhang [1], Fengchang Wu [3] and Qing Zhao [1,4,*]

[1] Key Laboratory of Pollution Ecology and Environmental Engineering, Institute of Applied Ecology, Chinese Academy of Sciences, Shenyang 110016, China; syzhang@iae.ac.cn (S.Z.); zhaobing17@mails.ucas.ac.cn (B.Z.); zhangxuejiao@iae.ac.cn (X.Z.)
[2] University of Chinese Academy of Sciences, Beijing 100049, China
[3] State Key Laboratory of Environmental Criteria and Risk Assessment, Chinese Research Academy of Environmental Sciences, Beijing 100012, China; wufengchang@vip.skleg.cn
[4] National-Regional Joint Engineering Research Center for Soil Pollution Control and Remediation in South China, Guangdong Key Laboratory of Integrated Agro-Environmental Pollution Control and Management, Institute of Eco-Environmental and Soil Sciences, Guangdong Academy of Sciences, Guangzhou 510650, China
* Correspondence: zhaoqing@iae.ac.cn
† These authors contributed equally to this work.

Text S1. Synthesis of PbS particles

Nanoscale PbS (nano-PbS) was prepared by mixing 0.379 g lead acetate, 0.152 g thioacetamide, and 0.364 g cetyltrimethylammonium bromine in 40 mL water. The mixture was stirred for 30 min, heated for 14 h at 120 °C, and then cooled to room temperature. Black solid products were separated via centrifugation at 3500 rpm for 20 min. Precipitates were washed three times with distilled water and ethanol, and then dried at room temperature for 5 h in a vacuum-dryer. The prepared nano-PbS was collected in sealed, oxygen-free centrifuge tubes.

Text S2. Analytical conditions and quality control of inductively coupled plasma–mass spectrometry (ICP-MS)

The working conditions were as follows. Sampling depth: 3.61 mm; cooling gas flow: 13.0 L/min; auxiliary gas flow: 0.60 L/min; atomization gas flow: 0.92 L/min; analysis pressure: 2.6×10^{-6} mbar; diffusion pressure: 1.8 mbar; atomization pressure: 2.68 bar; power: 1390 W; reflected power: 0.0 W; data acquisition method: scanning. The analysis time for each sample was 90 s. The detection limit was 0.1 μg/L, and the quantitation limit was 1 μg/L.

Before testing the sample, the ICP–MS was calibrated with the calibration solution. A series of Pb standard gradient dilution solutions were introduced into the instrument to form a linear relationship ($r > 0.9999$). Pb Standard samples were repeatedly measured every 20 experimental samples for calibration, and error values

were within ± 10%. The relative standard deviation (RSD) of the sample concentration was < 20%. Reagent and plant blank controls amended with distilled water were analyzed each time with the samples. Recoveries of the digestion and analytical procedures were evaluated by treating standard reference leaves (citrus and celery) following the same procedure.

Text S3. Analytical conditions of liquid chromatography–mass spectrometry (LC-MS)

Chromatographic separation was accomplished with a Thermo Vanquish system equipped with an ACQUITY UPLC® HSS T3 (150 × 2.1 mm, 1.8 μm, Waters) column maintained at 40 ˚C. The temperature of the autosampler was 8 ˚C. Gradient elution of analytes was carried out with 0.1% formic acid in water (A_2) and 0.1% formic acid in acetonitrile (B_2) or 5 mM ammonium formate in water (A_3) and acetonitrile (B_3) at a flow rate of 0.25 mL/min. Injection of 2 μL of each sample was carried out after equilibration. An increasing linear gradient of solvent B_2/B_3 (v/v) was used as follows: 0 ~ 1 min, 2% B_2/B_3; 1 ~ 9 min, 2% ~ 50% B_2/B_3; 9 ~ 12 min, 50% ~ 98% B_2/B_3; 12 ~ 13.5 min, 98% B_2/B_3; 13.5 ~ 14 min, 98% ~ 2% B_2/B_3; 14 ~ 20 min, 2% B_2-positive model (14 ~ 17 min, 2% B_3-negative model)[1].

The ESI-MSn experiments were executed with a Thermo Q Exactive mass spectrometer with a spray voltage of 3.5 kV and -2.5 kV in positive and negative modes, respectively. Sheath gas and auxiliary gas were set at 30 and 10 arbitrary units,

respectively. The capillary temperature was 325 ℃., respectively. The Orbitrap analyzer scanned over a mass range of m/z 81 ~ 1 000 for a full scan at a mass resolution of 70 000. Data-dependent acquisition (DDA) MS/MS experiments were performed with HCD scan. The normalized collision energy was 30 eV. Dynamic exclusion was implemented to remove some unnecessary information in MS/MS spectra[1].

Table S1. Characterizations of PbX_n (10 mg Pb/L) in pure water.

Treatment	Hydrodynamic size /nm	Zeta potential /mV
Nano-PbS	316 ± 6	-23 ± 1
Mic-PbO	1144 ± 70	20 ± 1

Table S2. Perturbed biological pathways in eggplant leaves exposed to nano-PbS.

	Perturbed pathway	*p*	Impact
1	Galactose metabolism	2.85×10^{-3}	0.085
2	Phenylpropanoid biosynthesis	3.18×10^{-3}	0.036
3	Phenylalanine metabolism	9.00×10^{-3}	0.058
4	ABC transporters	9.16×10^{-3}	0.058
5	Glycine, serine and threonine metabolism	2.19×10^{-2}	0.095
6	Tyrosine metabolism	2.57×10^{-2}	0.094
7	Phenylalanine, tyrosine and tryptophan biosynthesis	3.60×10^{-2}	0.056
8	Pentose phosphate pathway	3.87×10^{-2}	0.029
9	Glutathione metabolism	4.77×10^{-2}	0.097

Table S3. Perturbed biological pathways in eggplant leaves exposed to mic-PbO.

	Perturbed pathway	p	Impact
1	Galactose metabolism	1.89×10^{-4}	0.32
2	Tyrosine metabolism	2.06×10^{-3}	0.14
3	Linoleic acid metabolism	2.18×10^{-3}	0.36
4	ABC transporters	2.28×10^{-3}	0.14
5	Phenylalanine, tyrosine and tryptophan biosynthesis	6.95×10^{-3}	0.16
6	Phenylpropanoid biosynthesis	1.58×10^{-2}	0.11
7	Lysine degradation	1.85×10^{-2}	0.16

Table S4. Perturbed biological pathways in eggplant leaves exposed to $PbCl_2$.

	Perturbed pathway	p	Impact
1	Galactose metabolism	2.75×10^{-4}	0.32
2	Phenylalanine, tyrosine and tryptophan biosynthesis	4.43×10^{-4}	0.22
3	ABC transporters	1.55×10^{-3}	0.14
4	Alanine, aspartate and glutamate metabolism	2.77×10^{-3}	0.28
5	Phenylalanine metabolism	8.87×10^{-3}	0.26
6	Aminoacyl-tRNA biosynthesis	1.00×10^{-2}	0.16
7	Pyrimidine metabolism	1.54×10^{-2}	0.17
8	Butanoate metabolism	2.69×10^{-2}	0.19
9	Tryptophan metabolism	3.17×10^{-2}	0.15
10	Citrate cycle (TCA cycle)	4.92×10^{-2}	0.11

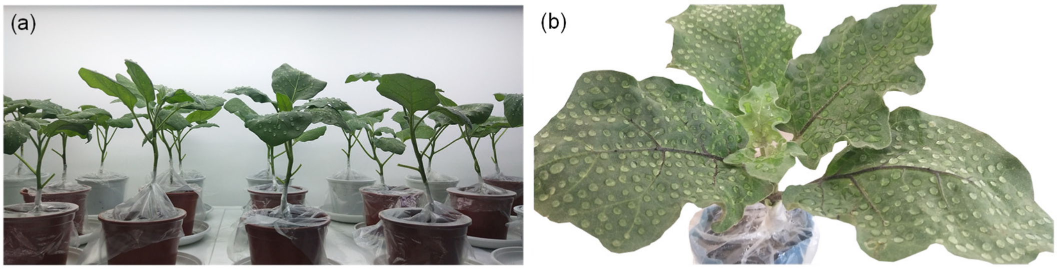

Figure S1. Plant experiment. (a) Photographs of the plants and (b) eggplant leaves after foliar application of 10 mg Pb/L dispersion.

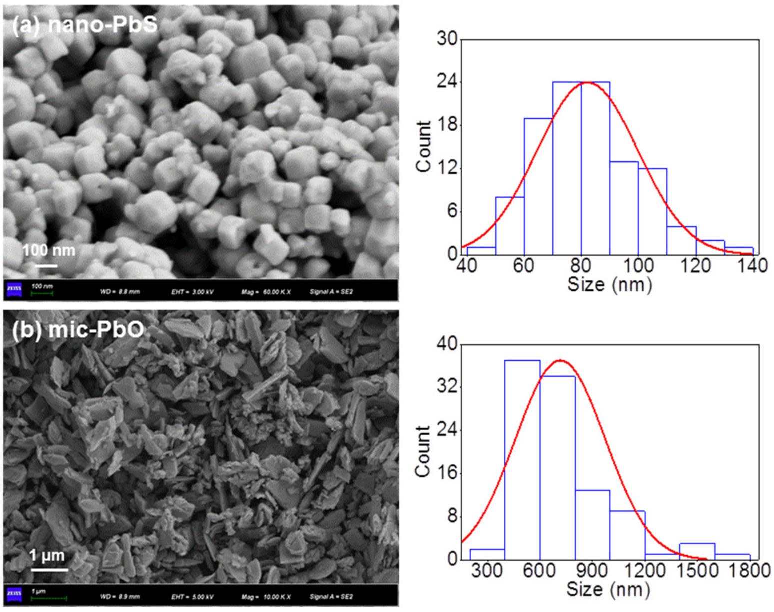

Figure S2. SEM images and size distributions of PbX_n. (a) nano-PbS; (b) mic-PbO.

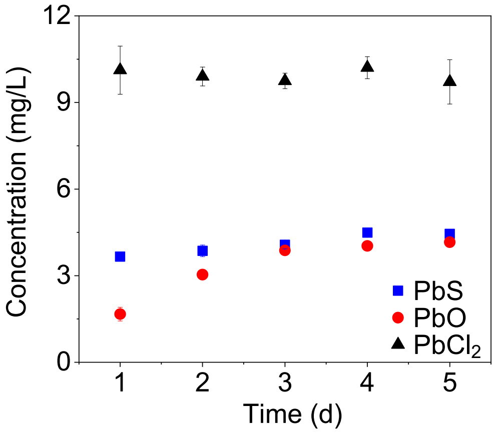

Figure S3. Concentrations of Pb ions in 10 mg Pb/L PbX_n suspensions and salt solutions. PbS: nano-PbS; PbO: mic-PbO.

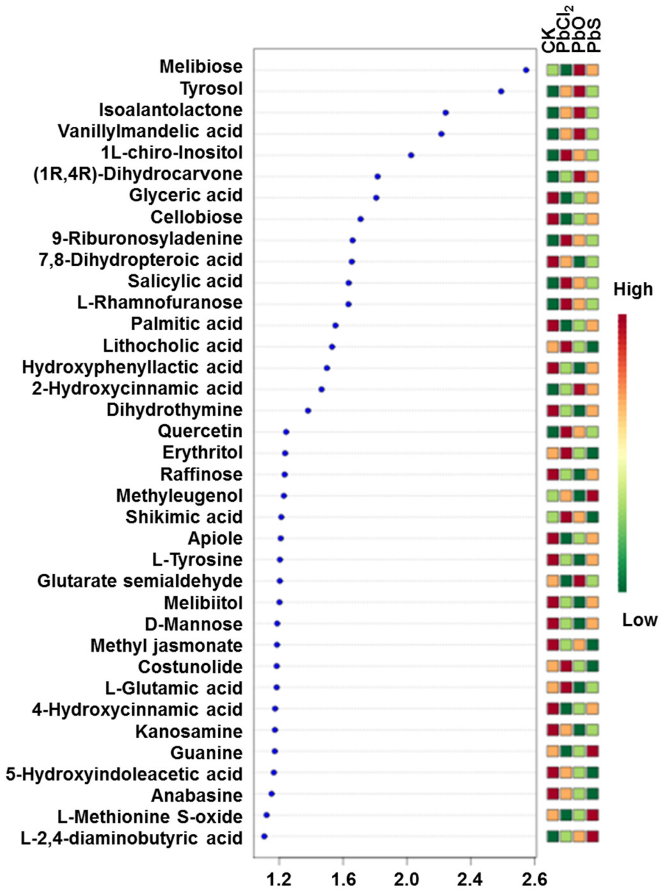

Figure S4. VIP scores from PLS-DA analysis of eggplant leaves showing the discriminating metabolites between unexposed control, nano-PbS, mic-PbO and $PbCl_2$. PbS: nano-PbS; PbO: mic-PbO; CK: control.

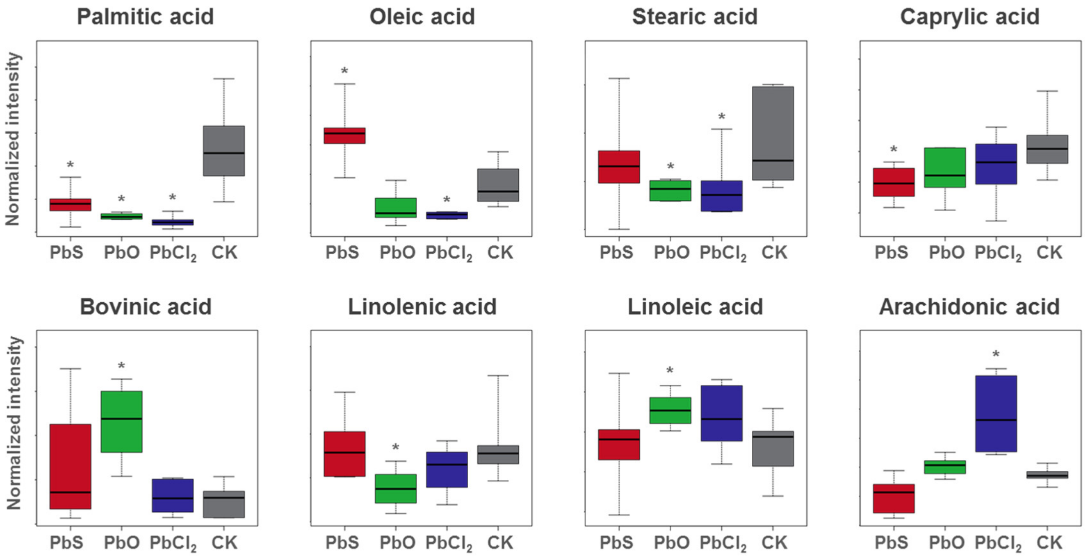

Figure S5. Box plots of the relative intensity of fatty acids in eggplant leaves after 5 days of PbX_n-treatments. PbS: nano-PbS; PbO: mic-PbO; CK: control.

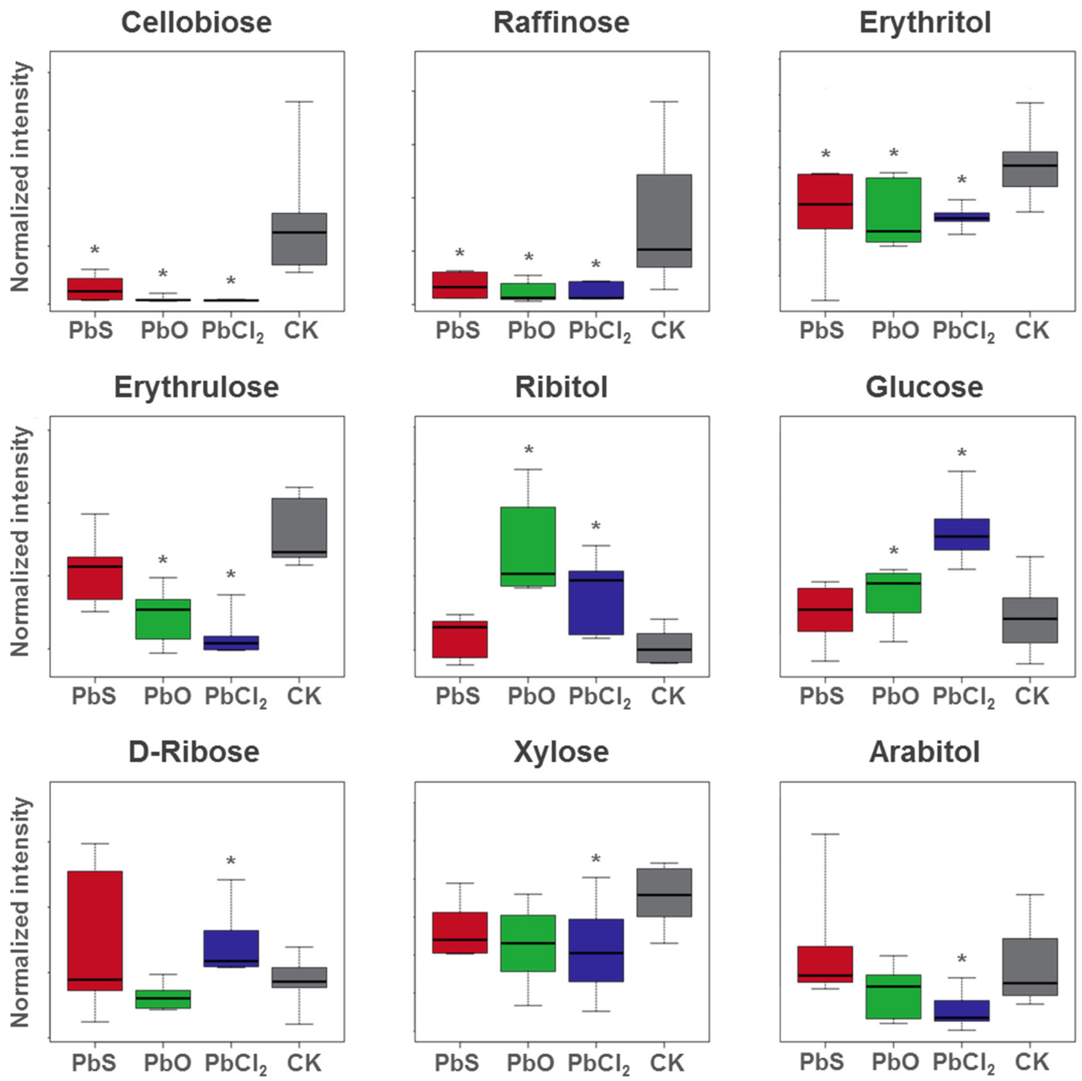

Figure S6. Box plots of relative intensity of sugars in eggplant leaves after 5 days of PbX_n-treatments. PbS: nano-PbS; PbO: mic-PbO; CK: control.

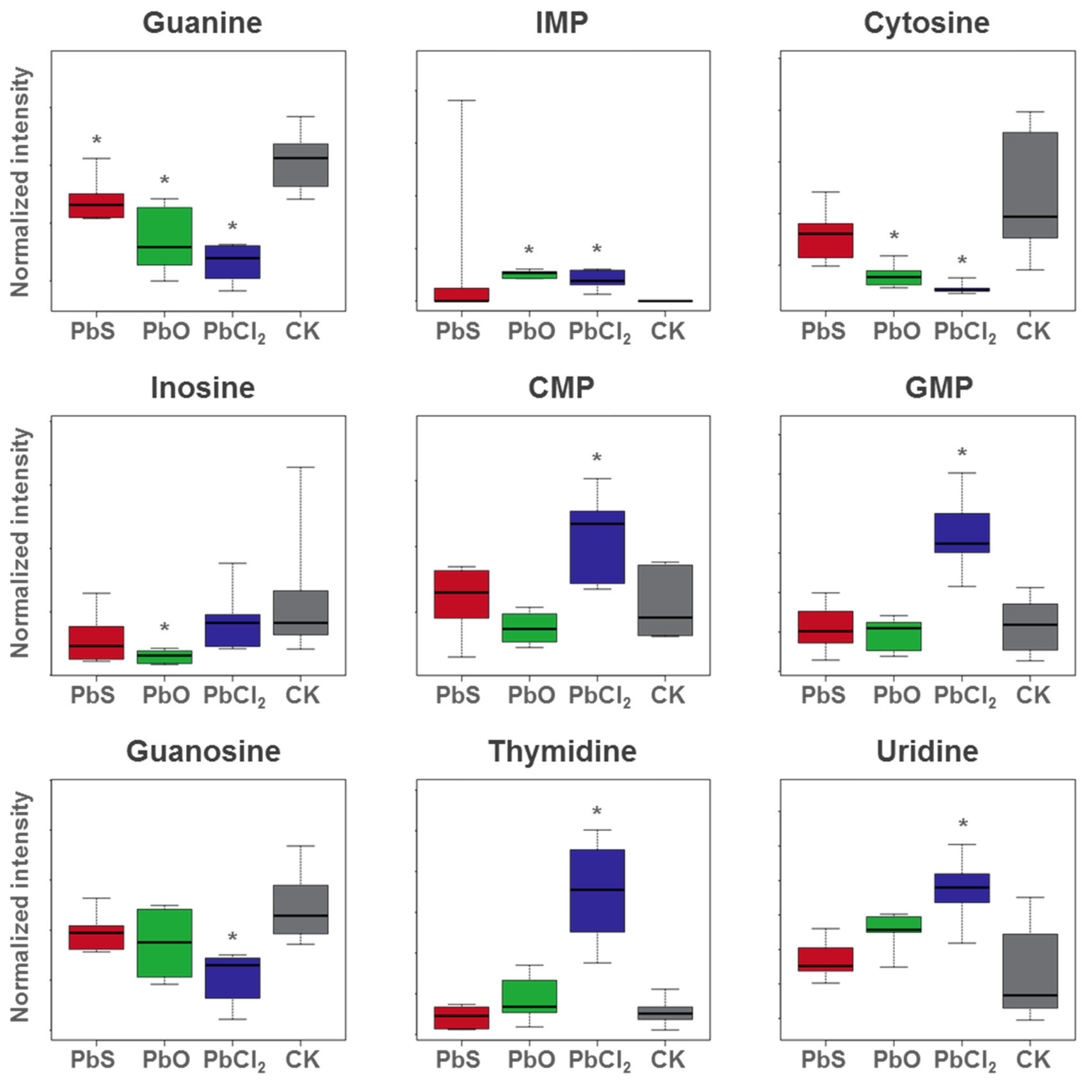

Figure S7. Box plots of relative intensity of nucleotide/side metabolites in eggplant leaves after 5 days of PbX_n-treatments. PbS: nano-PbS; PbO: mic-PbO; CK: control.

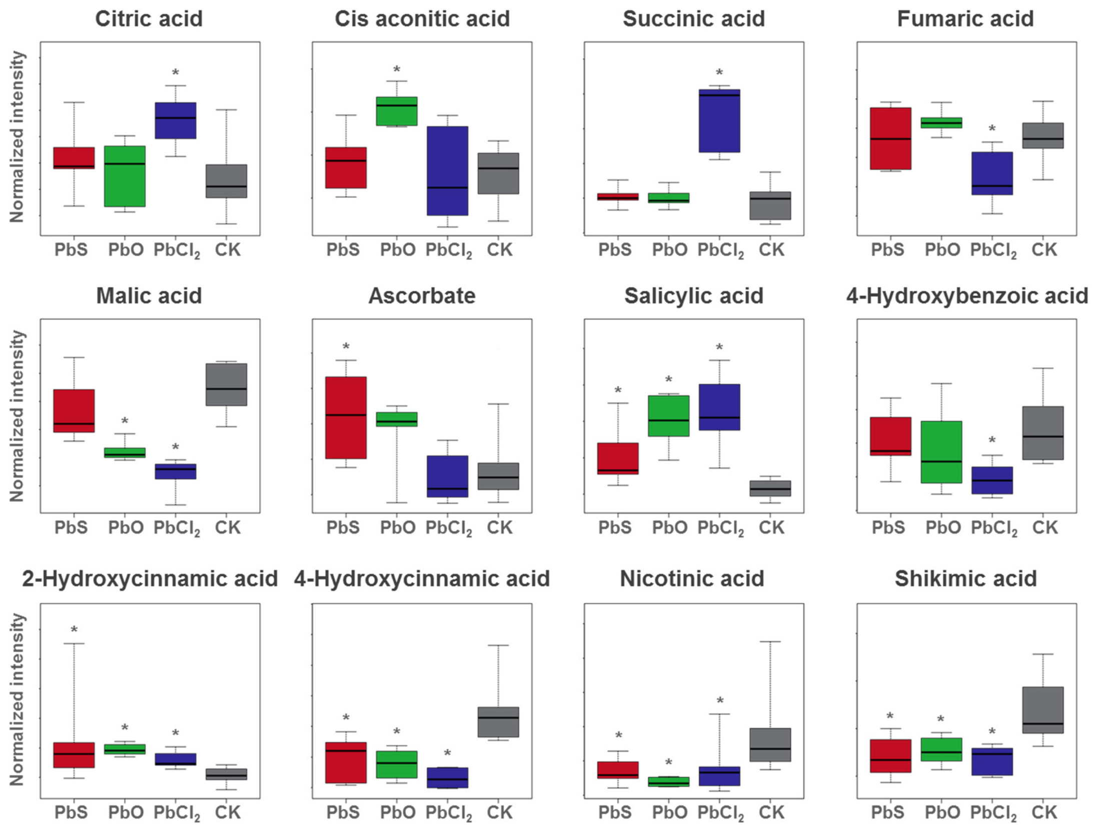

Figure S8. Box plots of relative intensity of organic acids and antioxidants in eggplant leaves after 5 days of PbX_n-treatments. PbS: nano-PbS; PbO: mic-PbO; CK: control.

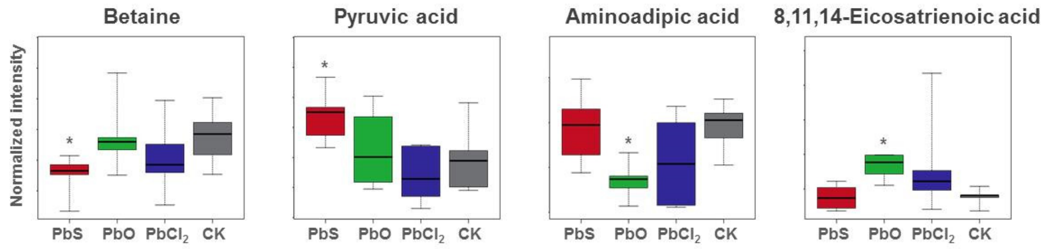

Figure S9. Box plots of relative intensity of metabolites in eggplant leaves after 5 days of PbX_n-treatments. PbS: nano-PbS; PbO: mic-PbO; CK: control.

References

1. Dunn, W. B.; Broadhurst, D.; Begley, P.; Zelena, E.; Francis-McIntyre, S.; Anderson, N.; Brown, M.; Knowles, J. D.; Halsall, A.; Haselden, J. N., Procedures for Large-Scale Metabolic Profiling of Serum and Plasma Using Gas Chromatography and Liquid Chromatography Coupled to Mass Spectrometry. *Nat. Protoc.* **2011,** *6* (7), 1060-1083.
